# Reduction of ventilator-associated pneumonia using the AnapnoGuard system

**DOI:** 10.1186/cc14292

**Published:** 2015-03-16

**Authors:** Y Bar-Lavie

**Affiliations:** 1 Rambam Medical Center, Haifa, Israel

## Introduction

Ventilator-associated pneumonia (VAP) is a common complication in mechanically ventilated patients. Frequently the pathogens responsible derive from aspirated secretions of the upper respiratory tract or the stomach. In order to prevent aspiration, two missions should be attained: a good tracheal cuff seal with a well-tolerated pressure, together with continuous evacuation of secretions from the subglottic space. These two goals can be achieved using the AnapnoGuard system and its related endotracheal tube (ETT).

## Methods

A single-center, open-label study in a general ICU. Control group: (retrospective data) mechanically ventilated patients on standard of care regular ETT, manual suction of the trachea and oral- pharyngeal space by nursing staff. Study group: (prospective data) connected at all times to the AnapnoGuard system: an ETT with two above-the-cuff suction ports and a third port and lumen for rinsing and CO_2_ measurement. A triple lumen harness is connected to a control system designed to measure CO_2_ levels above the cuff (to identify leaks), inflate the cuff accordingly, rinse and suction secretions above the cuff. To be included in the study patients had to have no pneumonia on admission and at least 3 days of mechanical ventilation. VAP was diagnosed for a new chest X-ray infiltrate accompanied by fever, leucocytosis and positive sputum culture. The study was approved by the hospital IRB.

## Results

Control group: 100 patients who received standard intubation and treatment in 2009 to 2010. Of these, four dropped out due screening failure of pneumonia on the day of enrollment. Study group: in 2011 to 2014, 192 patients were screened. Of these, 49 were found eligible and were enrolled. Of these, 14 dropped out (four screening failures with pneumonia on day of enrollment and 10 withdrawals with MV of less than 24 hours). Mean age was 51 (control) and 49 (study). Males were 75% of both groups and mean weight was 81 kg in both. VAP was diagnosed in 26 (27%) of controls and only three (8.5%) of the study group (*P *= 0.03). Mean time from admission to VAP diagnosis was 4.7 days in controls versus 5.12 in the study group (NS). No serious adverse events occurred.

## Conclusion

Patients connected to the AnapnoGuard system demonstrated a statistically significant lower VAP rate compared with the control group (8.5% vs. 27% respectively, *P *= 0.03). The estimated relative risk of VAP occurring in the control group was more than three times higher than the study group. Rinsing and aspiration of subglottic secretions combined with cuff pressure and seal management may be an effective method to prevent VAP.

